# Dendropanoxide Induces Autophagy through ERK1/2 Activation in MG-63 Human Osteosarcoma Cells and Autophagy Inhibition Enhances Dendropanoxide-Induced Apoptosis

**DOI:** 10.1371/journal.pone.0083611

**Published:** 2013-12-17

**Authors:** Ji-Won Lee, Kyoung-Sook Kim, Hyun-Kyu An, Cheorl-Ho Kim, Hyung-In Moon, Young-Choon Lee

**Affiliations:** 1 College of Natural Resources and Life Science, Dong-A University, Busan, South Korea; 2 Molecular and Cellular Glycobiology Unit, Department of Biological Sciences, SungKyunKwan University, Kyunggi-Do, South Korea; National Center for Scientific Research Demokritos, Greece

## Abstract

Anticancer effects of dendropanoxide (DP) newly isolated from leaves and stem of *Dendropanax morbifera* Leveille were firstly investigated in this study. DP inhibited cell proliferation and induced apoptosis in dose- and time-dependent manner in MG-63 human osteosarcoma cells, which was dependent on the release of cytochrome *c* to the cytosol and the activation of caspases. Moreover, the DP-treated cells exhibited autophagy, as characterized by the punctuate patterns of microtubule-associated protein 1 light chain 3 (LC3) by confocal microscopy and the appearance of autophagic vacuoles by MDC staining. The expression levels of ATG7, Beclin-1 and LC3-II were also increased by DP treatment. Inhibition of autophagy by 3-methyladenine (3-MA) and wortmannin (Wort) significantly enhanced DP-induced apoptosis. DP treatment also caused a time-dependent increase in protein levels of extracellular signal-regulated kinase 1 and 2 (ERK1/2), and inhibition of ERK1/2 phosphorylation with U0126 resulted in a decreased DP-induced autophagy that was accompanied by an increased apoptosis and a decreased cell viability. These results indicate a cytoprotective function of autophagy against DP-induced apoptosis and suggest that the combination of DP treatment with autophagy inhibition may be a promising strategy for human osteosarcoma control. Taken together, this study demonstrated for the first time that DP could induce autophagy through ERK1/2 activation in human osteosarcoma cells and autophagy inhibition enhanced DP-induced apoptosis.

## Introduction

Osteosarcoma is the most prevalent malignant bone tumor that occurs mainly in childhood and adolescence and the overall 5-year survival rate of osteosarcoma patients is 68% [1]. Despite the substantial improvement of survival rate by advances of adjuvant chemotherapy combined with surgery, the prognosis for patients with osteosarcoma still remains poor, owing to recurrent metastasis and the induction of drug resistance [2]. Thus, it is important to explore more effective chemotherapeutic agents for treating aggressive osteosarcoma. Moreover, chemotherapeutic agents currently used for cancer patients are known to have severe toxicity and significant side effects of chemotherapy [3]. To reduce chemotherapy-related side effects, natural compounds and their derivatives exerting their anticancer effects by inducing apoptosis have gradually gained considerable attention as a new source of chemotherapy [4].

It is well known that many chemotherapeutic drugs mainly exert their antitumor effect by inducing apoptosis in cancer cells and especially, apoptosis in cancer therapies is a crucial factor that affects sensitivity to chemotherapeutic agents [5], [6]. Furthermore, it has been recently reported that chemotherapeutic agents participate in killing cancer cells by triggering autophagy, called type II programmed cell death, which is a process of self-digestion that enables cells to cope with a variety of cellular stresses, such as nutrient starvation, ER stress, infection and hypoxia [7].

Recent studies have revealed that several natural products, including anthocyanins [8], voacamine [9], riccardin D [10], paclitaxel [11] and dihydroptychantol A [12], induce apoptosis and autophagy in human osteosarcoma cells. These studies have demonstrated that they induce autophagy preceding apoptosis and autophagy inhibition by its inhibitor enhances apoptosis in cells treated with them.

*Dendropanax morbifera* Leveille (Araliaceae) is an endemic species growing in the south-western part of South Korea and has been used in folk medicine for the treatment of headache, infectious diseases and skin diseases. More recently, we have shown that oleifolioside A, a cycloartane-type glycoside isolated from the lower stem of *D. morbifera,* induced a caspase-independent apoptosis in human cervical carcinoma HeLa cells, which was caused by the increase of the pro-apoptotic Bcl-2 member proteins, resulting in a loss of mitochondrial membrane potential and the release of cytochrome *c* from mitochondria, leading to mitochondrial release of AIF and EndoG and their translocation to the nucleus [13]. In addition, we have also demonstrated that oleifolioside A suppresses LPS-stimulated iNOS and COX-2 expression through the down-regulation of NF-κB and MAPK activities in RAW 264.7 macrophages [14].

Recently, we have also isolated a new compound, dendropanoxide (DP), from leaves and stem of *Dendropanax morbifera* Leveille, which has anti-diabetic effects in streptozotocin-induced diabetic rats [15]. However, the inhibitory effects of DP on cancer cells and its underlying molecular mechanisms have never been studied. Therefore, we have attempted to elucidate the possible biological mechanisms controlling the anti-tumor activity of DP.

In this study, we have investigated for the first time the anticancer effects of DP on human osteosarcoma cells and have sought to clarify the precise mechanism of its action. We firstly showed that DP induces autophagy and apoptosis in MG-63 human osteosarcoma cells, and apoptosis is enhanced by inhibition of autophagy.

## Materials and Methods

### Materials

Monodansylcadaverine (MDC), 4’,6-diamidino-2-phenylindole dihydrochloride (DAPI) and 3-methyladenine (3-MA) were purchased from Sigma-Aldrich. (St. Louis, MO, USA). Wortmannin, SB203580, SP600125 and Z-VAD-FMK were obtained from Calbiochem (Darmstadt, Germany). The ERK1/2 inhibitor U0126, was purchased from Promega (Madison, WI, USA). Antibodies for ATG7, Beclin-1, LC3, p-p38, p38, p-JNK, JNK, caspase-8, caspase-9 and caspase-3 were purchased from Cell Signaling Technology (Dancers, Mass, USA); antibodies for p-ERK, ERK and cytochrome *c* were obtained from Santa Cruz Biotechnology (CA, USA); antibody for glyceraldehyde-3-phosphate dehydrogenase (GAPDH) was purchased from Millipore (Milford, MA, USA); antibody for β-actin was obtained from Sigma-Aldrich. (St. Louis, MO, USA); Horseradish peroxidase (HRP)-conjugated secondary antibodies were purchased from Enzo Life Science (Farmingdale, NY, USA); Fluorescein (FITC)-conjugated antibody was purchased from Vector Laboratories (CA, USA). Annexin V-FITC apoptosis detection kit and Bradford protein assay kit were obtained from BD Biosciences (San Jose, CA) and Bio-Rad Laboratories (Hercules, CA), respectively. Dendropanoxide (DP) isolated from leave of *D. morbifera* was prepared as described previously [15], dissolved in dimethyl sulfoxide (DMSO) as a stock solution at 60 mM concentration, and stored in aliquots at −20°C.

### Cell cultures

Human osteosarcoma cancer cell line MG-63, human glioblastoma cell line T98G and human hepatocellular carcinoma cell line Hep3B were obtained from American Type Culture Collection (Rockville, MD, USA). The cells were grown in Dulbecco’s modified Eagle’s medium (DMEM; WelGENE Co., Daegu, Korea) supplemented with 10% (v/v) heat-inactivated fetal bovine serum (FBS), 100 U/ml penicillin, and 100 µg/ml streptomycin at 37°C under 5% CO_2_.

### Cell viability

Cell viability assay was performed using a similar procedure as described previously [13]. Briefly, cells were plated in 24-well culture plate (2×10^4^ cells/well). After 24 h, the cells were treated with various concentrations of DP. After DP treatment for 12 and 24 h, cells were washed with PBS and MTT (0.5 mg/ml) was added to each. After incubation for 3 h, DMSO was added to dissolve the formazan crystal from MTT reduction and the amount of formazan salt was determined by measuring the OD at 590 nm using an ELISA plate reader (Bio-rad, Hercules, CA, USA). Cell viability was quantified as a percentage compared to the control.

### Immunoblot analysis

Immunoblot analysis was performed as previously described[13]. Cell lysate were separated in SDS-polyacrylamide gel electrophoresis and transfer to PVDF membrane. Membranes were blocked with 5% skim milk and incubated with corresponding antibody against a specific protein, and then with an appropriate horseradish peroxidase conjugated secondary antibody. The detection of specific proteins was carried out with an enhanced chemiluminescent detection system. The densitometric signal intensity of each band was analyzed with a Scion Image Instrument (Scion Co., Frederick, MD, USA).

### Immunostaining

MG-63 cells were plated on sterile coverslips and treated with DP, fixed with 3% paraformaldehyde (PFA) for 10 minutes at 37°C. After fixation, the cells were permeabilized with cold 100% methanol at −20°C for 10minutes and then blocked with 1% BSA for 1 h at 37°C, followed by incubation for overnight at 4°C with the anti-LC3 antibody, and then cells were reacted with FITC-conjugated secondary antibody for 1 h at 37°C. The nucleus was stained with DAPI for 5 min at 37°C. For fluorescence imaging were then obtained using LSM 700 confocal laser scanning microscope (Carl Zeiss, Oberkochen, Germany).

### MDC staining

After DP treatment, cells were fixed with 3% PFA solution in PBS at 37°C for 10 min, and then cells were stained by monodansylcadaverine (MDC), an autofluorescent dye that accumulated in autophagic vacuole, by incubating cells with 0.05 mM MDC at 37°C for 10 min. After incubation, cells were analyzed using an Olympus BX51 fluorescence microscope (Center Valley, PA, USA).

### Annexin V and PI staining

After treatment with the indicated concentrations of DP for 12 and 24 h, MG-63 cells were harvested by trypsin-EDTA and stained with Annexin V-FITC and propidium iodide (PI) according to the manufacture’s instructions. After staining, cells were analyzed with Beckman-Coulter Cytomics FC500 flow cytometer (Beckman-Coulter, Miami, FL, USA).

### Statistical analysis

All experiments data are expressed as means ± SEM of at least three replicates in each group. Comparisons between the two groups were determined by Student’s *t*-test and * *P*<0.05 was considered significant difference.

## Results

### Effect of DP on cell viability of human cancer cells

**Figure 1:**
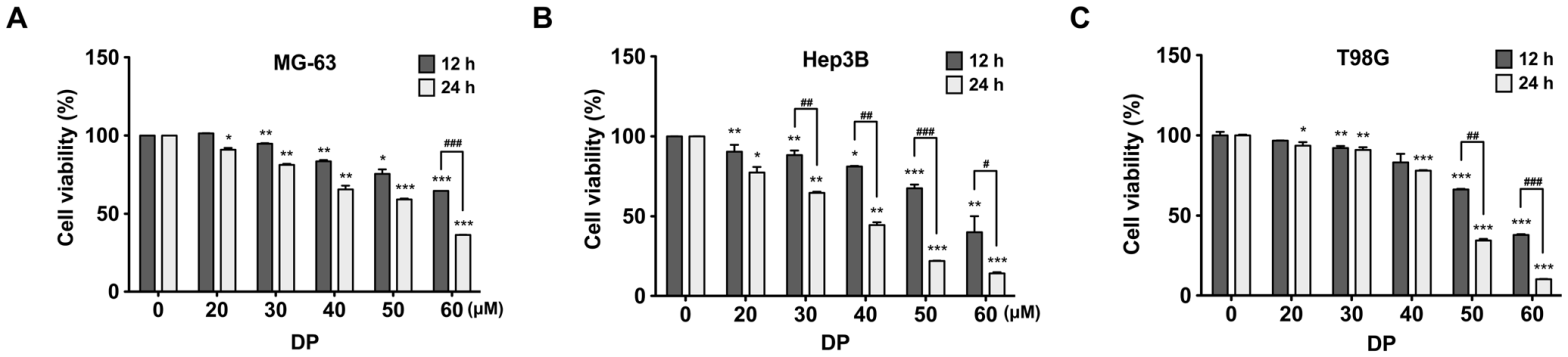
Effects of DP on cell viability in human cancer cells. (A) MG-63, (B) Hep3B and (C) T98G cells were treated with various concentrations of dendropanoxide (DP) for 12 and 24 h and then cell viability were measured by MTT assay. Data were presented as percentage of control and were mean ± SEM (n = 3−4). ^*^P<0.05, ^**^P<0.01 and ^***^P<0.001 compare with control; ^#^P<0.05 ^##^P<0.01 and ^###^P<0.001 compared with DP.

To investigate the effects of DP on cell viability of MG-63 human osteosarcoma cells, cells were treated with various concentration of DP for 12 h or 24 h, and then subjected to MTT assays. As shown in Fig. 1A, DP treatment induced cell growth inhibition in a dose- and time-dependent manner; DP treatment at the concentration of 50 µM for 24 h inhibited the cell proliferation by 37%, while DP treatment with 60 µM showed a 57% inhibition effect. However, DP treatment for 12 h at the concentration of 60 µM resulted in 31% inhibition of cell viability. To evaluate whether the inhibition of cell growth by DP is specific to MG-63 cells, or a more general effect, the same experiment was conducted in human hepatoma Hep3B and human glioblastoma T98G cells. We also observed the inhibition of cell growth by DP in Hep3B and T98G cells, even though the effective concentration of DP used in two cell lines was different (Fig. 1B and C).

### DP induces apoptosis in MG-63 cells

**Figure 2:**
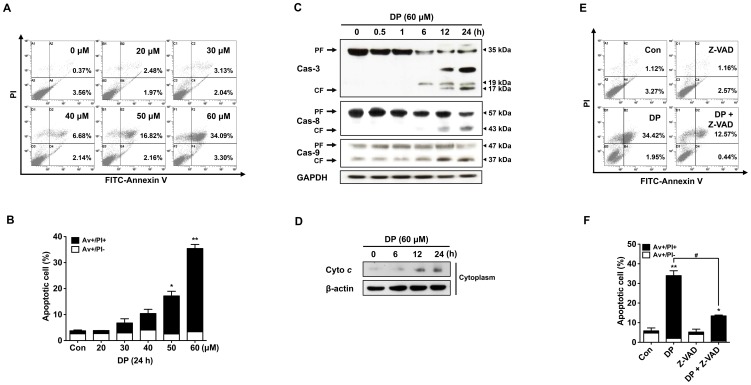
DP induced both extrinsic and intrinsic apoptosis pathway in MG-63 cells. After treatment with various concentration of dendropanoxide (DP) for 24 h and then apoptotic cells were detected by Annexin V and PI stained (A) and quantitative analysis of Av+/PI- (early apoptosis) and Av+/PI+ (late apoptosis) cells (B) by flow cytometry. Cells were treated with 60 µM DP for different times and then analyzed by immunoblotting with an antibody against caspase-3 (Cas-3), caspase-8 (Cas-8) and caspase-9 (Cas-9) (C). An accumulation of cytochrome *c* (Cyto *c*) in the cytosolic fraction was analyzed by immunoblotting (D). Cells were treated with 60 µM DP for 24 h in the presence or absence of 40 µM Z-VAD-FMK, the percentage of apoptotic cells were detected by flow cytometry for Annexin V/PI staining (E). Quantitative determination of Av+/PI- and Av+/PI+ MG-63 cells by flow cytomertry (F). Data were expressed as the mean ± SEM of repeated three times experiments. ^*^P<0.05 and ^**^P<0.01 compare with control; ^#^P<0.05 compared with DP.

To examine whether DP inhibits the proliferation of MG-63 cells by inducing apoptosis, the percentage of apoptotic cells was determined by Annexin V-PI double staining after a 24-h treatment with various concentrations of DP. Annexin V-FITC binds to exposed phosphatidylserine on apoptotic and necrotic cells, and PI staining exhibits entry into the late apoptotic cells and necrotic cells. As shown in Fig. 2A and B, MG-63 cells positive for Annexin V and Annexin V-PI were significantly increased in a dose-dependent manner, demonstrating the apoptotic effect of DP on MG-63 cells. It is well-known that caspase-3, a key protease in the apoptotic machinery, is activated by cleavage into two smaller subunits by caspase-8 and/or caspase-9 when the cells undergo apoptosis after synthesis as procaspase-3 [16], [17]. Thus, the effect of DP on the induction of apoptosis through the activation of caspase-3, -8 and -9 in MG-63 cells was investigated by immunoblotting with caspase antibodies. The cleaved forms (17 and 19 kDa) of caspase-3 were firstly detected after a 6-h treatment with 60 µM DP and then gradually increased (Fig. 2C). Furthermore, the cleaved forms (43 and 37 kDa) of caspase-8 and-9 were also clearly detected after a 12-h treatment with 60 µM DP (Fig. 2C). To investigate whether the activation of caspase-9 by DP was dependent on the release of cytochrome *c* from mitochondria to cytosol, we examined the cytochrome *c* release by immunoblotting with its antibody. As shown in Fig. 2D, the cytochrome *c* release from mitochondria to cytosol was firstly detected after a 6-h treatment with 60 µM DP and then gradually increased. To further confirm caspase-dependent apoptosis by DP, we checked the percentage of apoptotic cells by Annexin V-PI double staining after a 24-h treatment with caspase inhibitor. As shown in Fig. 2E and F, pretreatment of cells with the pan-caspase inhibitor Z-VAD-FMK significantly decreased the percentage of apoptotic cells as compared to DP treatment alone. These results indicate that DP induces apoptosis through the activation of caspases and the release of cytochrome *c* to the cytosol in MG-63 cells.

### DP also induces autophagy in MG-63 cells

**Figure 3:**
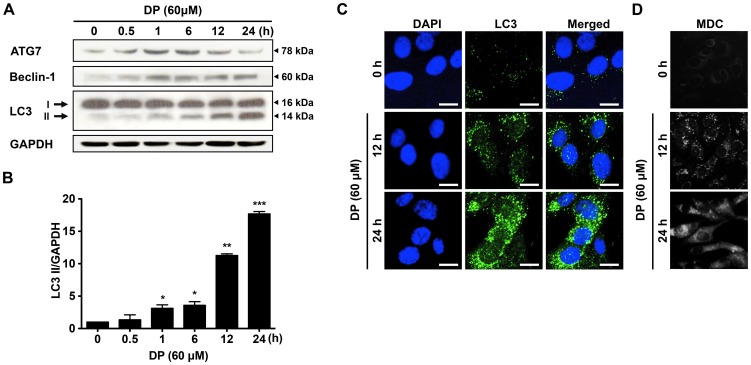
Effects of DP on autophagy in MG-63 cells. For representative immunoblot analysis of ATG7, Beclin-1 and LC3-II protein expression levels, cells were treated with 60 µM DP for different times (A). (B) Densitometric analysis of LC3-II/GAPDH in Fig. 3A. MG-63 cells were treated with 60 µM DP for 12 and 24 h, after fixation, cells were immunostained with anti-LC3 antibodies (FITC; green). Nuclei were stained with DAPI (blue), and analyzed by confocal microscopy (scale bars: 20 µm) (C). Autophagic vacuoles were stained by MDC (bright color), and observed by fluorescence microscopy (D). Results are shown as the mean ± SEM of repeated 3 times experiments. ^*^ *P*<0.05, ^**^ *P*<0.01 and ^***^ *P*<0.001 compared with control.

It was recently reported that several natural products induced apoptosis and autophagy in human osteosarcoma cells [8−12]. To investigate whether DP induces autophagy in MG-63 cells, the changes in three autophagy-related marker proteins, Atg7, Beclin-1 and microtubule-associated protein 1 light chain 3-II (LC3-II), were analyzed by immunoblotting. As shown in Fig. 3A and B, the expression levels of ATG7, Beclin-1 and LC3-II were increased after 1 h of DP treatment compared to control. Especially, DP induced a significant increase of LC3-II in a time-dependent way which began at 12 h and peaked at 24 h. As a common method for detecting autophagy, localization of LC3 to the autophagosome can be visualized as puncta accumulation by fluorescence microscopy [18]. Therefore, we examined the intracellular localization of LC3 in autophagic vacuoles induced by DP in MG-63 cells by immunofluorescence analysis using fluorescent antibodies to LC3. As shown in Fig. 3C, specific punctate distribution of endogenous LC3 were observed as punctate dots of green fluorescence after 12 h by DP treatment compared to that of control, and LC3 puncta formation was abundant in cells treated for 24 h. These results indicate that DP treatment induces LC3-associated autophagosome formation in MG-63 cells. To further ascertain the autophagy formation in DP-treated cells, the formation of autophagosome and autophagic vacuoles was examined by MDC staining which is generally used to detect autophagic vacuoles. The accumulation of MDC-positive vesicles by DP treatment was increased in a time-dependent manner, verifying the activation of autophagy by DP (Fig. 3D). Taken together, these results clearly indicate that DP induces autophagy in MG-63 cells.

### Inhibition of autophagy enhances DP-induced apoptosis of MG-63 cells

**Figure 4:**
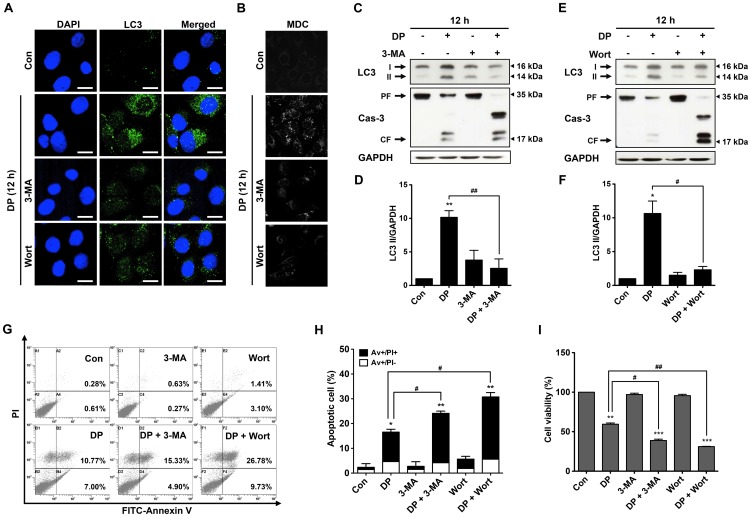
Inhibition of DP-induced autophagy enhanced apoptosis in MG-63 cells. Cells were treated with 60 µM dendropanoxide (DP) for 12 h in the presence or absence of 10 mM 3-MA and 10 µM Wort, after fixation, cells were immunostained with anti-LC3 antibodies (FITC; green). Nuclei were stained with DAPI (blue) and analyzed by using confocal microscopy (scale bars: 20 µm) (A). Autophagic vacuoles were labeled with MDC (bright color) and detected by fluorescence microscopy (B). Representative immunoblot analysis of LC3-II and caspase-3 (Cas-3) protein expression levels (C) and (E). (D) and (F) Densitometric analysis of LC3-II/GAPDH in Fig. 4C and E. The percentage of apoptotic cell was analyzed by flow cytometry for Annexin V/PI staining (G). Quantitative determination of Av+/PI- and Av+/PI+ MG-63 cells by flow cytomertry (H). Cell viability was measured by MTT assay (I). Data were expressed as the mean ± SEM of repeated 3 times experiments. ^*^ *P*<0.05, ^**^ *P*<0.01 and ^***^ *P*<0.001 compare with control; ^#^ *P*<0.05 and ^##^ *P*<0.01 compared with DP.

Recent studies have demonstrated that autophagy inhibition by its inhibitor can enhance apoptosis in human osteosarcoma cells treated with natural products [8], [11], [12]. To investigate the effect of autophagy on cell apoptosis in DP-treated MG-63 cells, therefore, wortmannin (Wort) and 3-methyladenine (3-MA), two kinds of specific autophagy inhibitors, were used to suppress the autophagy induced by DP treatment. The inhibitory effects of 3-MA and Wort on specific punctate distribution of endogenous LC3 and the formation of autophagic vacuoles in DP-treated MG-63 cells were investigated by immunofluorescence analysis and MDC staining, respectively. As shown in Fig. 4A and B, The subcellular distribution of LC3 and formation of MDC-positive autophagosomes induced by DP treatment were significantly inhibited by 3-MA and Wort. Next, the changes in the protein levels of caspase-3 and LC3-II after DP treatment in the presence of 3-MA and Wort were investigated by immunoblotting. As shown in Fig. 4C to F, the accumulation of LC3-II after DP treatment was suppressed in the presence of 3-MA and Wort, but the cleavage of caspase-3 after DP treatment was enhanced. DP treatment with Wort exhibited greater effect in the cleavage of caspase-3 than that with 3-MA. To confirm the effect of 3-MA and Wort on the induction of apoptosis, the percent of apoptotic cells was determined by FACS analysis following Annexin V-PI double staining. As shown in Fig. 4G and H, MG-63 cells positive for Annexin V and Annexin V-PI were significantly increased in DP-treated cells in the presence of 3-MA and Wort, compared with those treated with DP alone. Furthermore, DP treatment in the presence of 3-MA and Wort for 12 h significantly reduced cell viability as compared to DP treatment alone (Fig. 4I). These results clearly indicate that inhibition of autophagy by 3-MA or Wort could enhance DP-induced apoptosis in MG-63 cells.

### DP induces autophagy through activation of ERK1/2 pathway

**Figure 5:**
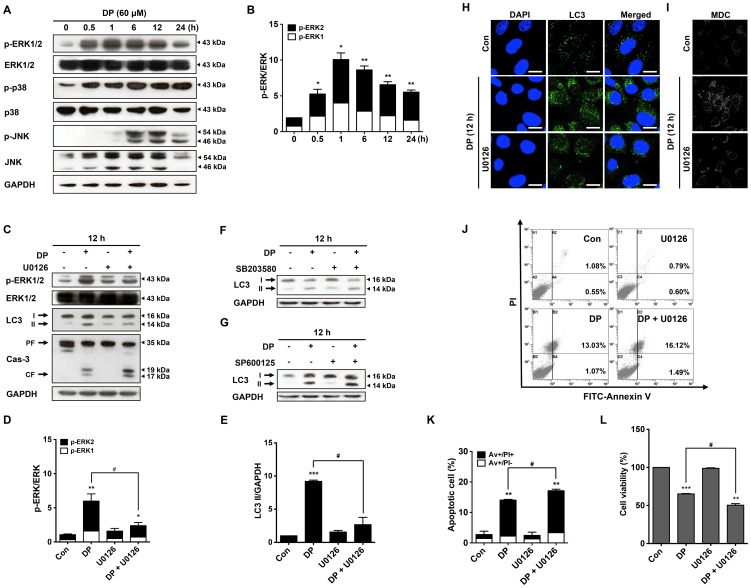
DP induced autophagy through ERK1/2 signaling pathway. For representative immunoblot analysis of p-ERK1/2, ERK1/2, p-p38, p38, p-JNK and JNK protein expression levels, cells were treated with 60 µM dendropanoxide (DP) for different times (A). (B) Densitometric analysis of p-ERK/ERK in Fig. 5A. MG-63 cells were treated with 60 µM DP for 12 h in the presence or absence of 50 µM U0126 and then protein expression levels of p-ERK1/2, ERK1/2, LC3-II and caspase-3 were analyzed by immunoblotting (C). (D) Densitometric analysis of p-ERK/ERK in Fig. 5C. (E) Densitometric analysis of LC3-II/GAPDH in Fig. 5C. Cells were incubated with 60 µM DP for 12 h in the presence or absence of 20 µM SB203580 and then protein expression level of LC3-II was analyzed by immunoblotting (F). For representative immunoblot analysis of LC3-II protein expression levels, cells were treated with 60 µM DP for 12 h in the presence or absence 10 µM SP600125 (G). MG-63 cells were incubated with 60 µM DP for 12 h in the presence or absence 50 µM U0126, after fixation, the nuclei were stained with DAPI (blue), and the distribution of endogenous LC3 (anti-LC3 FITC; green) was detected by confocal microscopy (scale bars: 20 µm) (H) . Autophagic vacuoles were stained with MDC (bright color), and observed with fluorescence microscopy (I). The percentage of apoptotic cell was evaluated by flow cytometry using Annexin V/PI staining (J). Quantitative analysis of Av+/PI- and Av+/PI+ MG-63 cells by flow cytomertry (K). Cell viability was determined by MTT assay (L). Results are shown as the mean ± SEM of repeated 3 times experiments. ^*^P<0.05, ^**^P<0.01 and ^***^P<0.001 compare with control; ^#^P<0.05 compared with DP.

It has been recently reported that induction of autophagy depends on the activation of ERK1/2 [19−22]. To investigate whether the activation of ERK1/2 contributes to the induction of autophagy, therefore, the expression level of the phosphorylated form of ERK1/2 was examined by immunoblotting. As shown in Fig. 5A and B, DP treatment induced a significant activation of ERK1/2 in a time-dependent way which began at 30 min and peaked at 1 h and then gradually decreased. Also, DP treatment increased the expression level of the phosphorylated forms of JNK and p38 MAPK (Fig. 5A), but their chemical inhibitors failed to inhibit DP-induced autophagy (Fig. 5F and G). To further confirm the effect of ERK1/2 activation on autophagy, ERK1/2 specific inhibitor U0126 was used to inactivate ERK1/2. DP treatment with U0126 significantly attenuated the activation of ERK1/2 and LC3-II accumulation, but significantly increased the protein level of cleaved form of caspase-3 (Fig. 5C-E). In addition, distribution of endogenous LC3 and MDC-positive autophagosomes formation were also reduced after the combination treatment of U0126 and DP (Fig. 5H and I). Although U0126 itself didn’t affect apoptosis and cell viability, furthermore, the combination treatment of U0126 and DP increased the apoptotic cell death, but inhibited the cell proliferation by 50% (Fig. 5J-L). These results indicate that DP-induced autophagy is regulated by ERK1/2 signaling pathway, and inhibition of autophagy by U0126 enhances DP-induced apoptosis in MG-63 cells.

## Discussion

Although the precise role of autophagy in both cell survival and cell death is still very controversial [23], [24], accumulating evidence indicates that natural compounds from plants induce cancer cell death by regulation of autophagy [25]. More recent studies have revealed that some natural products, such as anthocyanins, voacamine, riccardin D, paclitaxel and dihydroptychantol A, induce apoptosis and autophagy in human osteosarcoma cells [8]-[12]. In the present study, we have demonstrated for the first time that dendropanoxide (DP), a new compound isolated from *Dendropanax morbifera* Leveille, also induce autophagy and apoptosis in human osteosarcoma cells. Our results revealed the DP could inhibit cell proliferation and induce apoptotic cell death in MG-63 cells, which was dependent on the release of cytochrome *c* to the cytosol and the activation of caspases. The induction of autophagy by DP treatment was also evidenced by the formation of autophagosomes, a punctuate pattern of LC3 immunostaining and the accumulation of biochemical hallmark proteins of autophagy (ATG7, Beclin-1 and LC3-II), as shown in the previous studies [8−12].

ATG7, Beclin-1 and LC3-II are known to play pivotal roles in the formation of autophagosomes [24], . Especially, ATG7 is reported to be essential for ATG12 conjugation, LC3 modification systems, and autophagosome formation in mammals [26]. The conversion of LC3-I to LC3-II correlates with the extent of autophagosome formation and thus, the amount of LC3-II is the most widely used biomarker of autophagosomes formation [28]. In this study, we demonstrated that DP increases the expression levels of ATG7, Beclin-1 and LC3-II in a time-dependent manner in MG-63 cells, as evidenced by immunoblotting. This finding is consistent with a recent report [22] that aristolochic acid I (AAI), a natural compound from plant, induced a time-dependent accumulation of ATG7, Beclin-1 and LC3-II in NRK52E renal tubular epithelial cells, and small-interfering RNA knockdown of Beclin-1 gene or ATG7 gene resulted in a decrease of LC3-II formation induced by AAI, which was accompanied by an enhanced apoptosis. Furthermore, we found here that DP treatment induced a remarkable increase of MDC incorporation and this effect was almost completely inhibited by pretreatment with specific autophagy inhibitors, 3-MA and wortmannin.

Whether or not the process of autophagy regulates directly the process of apoptosis, or they only share some common machinery remains unclear at present [23], but various anticancer chemotherapies have been shown to induce autophagy in cooperation with apoptosis to induce cell death [7], . In addition, recent studies have indicated that apoptosis induced by chemotherapeutic agents could be promoted by autophagy inhibition [7], [23], [24]. In this study, we have also demonstrated that inhibition of autophagy by 3-MA and wortmannin significantly enhanced DP-induced apoptosis in MG-63 cells, whereas wortmannin and 3-MA themselves didn’t affect cell apoptosis, which was evidenced by increased cleavage of caspase-3 and decreased levels of a punctuate pattern of LC3, LC3-II accumulation, formation of MDC-positive autophagosomes, and cell viability. These results are in agreement with recent studies [11], [12] that inhibition of autophagy by 3-MA significantly increased paclitaxel-induced apoptosis in Saos-2 human osteosarcoma cells [11] and dihydroptychantol A-induced apoptosis in U2OS human osteosarcoma cells [12]. Collectively, these results suggest that autophagy could protect human osteosarcoma cells from apoptosis induced by natural compounds.

It is known that autophagy process is tightly regulated by a cascade of kinases [21] and accumulating evidences indicated that autophagy in various cancer cells could be induced through the activation of ERK1/2 by natural compounds, including AAI [22], daunorubicin [29], curcumin [30] and triterpenoid B-group soyasaponins [31]. In this study, we found that DP actually activated ERK1/2 and ERK activation preceded autophagy induction, as shown in the previous studies [31], [32]. Moreover, inhibition of ERK1/2 by U0126 significantly reduced the induction of autophagy, which leads to increase the cytotoxic effect of DP, as evidenced by the same results as shown in 3-MA and wortmannin, suggesting that DP induces autophagy through activation of ERK1/2 pathway. On the other hand, it is noticeable that although DP increased the phosphorylations of JNK and p38 MAPK, their chemical inhibitors could not block DP-induced autophagy. Similar to our results, a recent report revealed that AAI induced autophagy in NRK52E cells via the ERK1/2 pathway, the activation of JNK and p38 MAPK was not observed in AAI-induced NRK52E cells [22]. Further studies are required to understand the signalling pathways involved in the induction of autophagy by exposure to various compounds in numerous cancer cell types.

In conclusion, our study demonstrated for the first time that DP treatment in human osteosarcoma MG-63 cells leads to induction of autophagy that is modulated by ERK1/2 pathway and autophagy inhibition results in enhanced DP-induced apoptosis. Our findings will provide a new drug candidate as an alternative therapy for human osteosarcoma in the future.
